# Results of cement spacer sonication in the second stage of two-stage treatment of shoulder arthroplasty infection

**DOI:** 10.1186/s13018-018-0763-8

**Published:** 2018-03-20

**Authors:** Carlos Torrens, Fernando Santana, Lluis Puig, Luisa Sorli, Albert Alier

**Affiliations:** 1 Department of Orthopedics, Hospital del Mar. Parc de Salut Mar, Passeig Marítim 25-29, 08003 Barcelona, Spain; 2 Service of Internal Medicine and Infectious Diseases, Hospital del Mar. Parc de Salut Mar, Passeig Marítim 25-29, 08003 Barcelona, Spain

**Keywords:** Shoulder arthroplasty, Infection, Two-stage treatment and sonication

## Abstract

**Background:**

The objective of this study is to present the results of cement spacer sonication in the second stage of two-stage treatment of shoulder arthroplasty infection and to determine the rate of positive cultures in the second-stage surgery in shoulder arthroplasty and its meaning.

**Methods:**

Twenty-one patients (22 cement spacers) treated with two-stage surgery because of a shoulder arthroplasty infection were included. In the second stage, the cement spacer was sent for sonication and at least four tissue cultures were obtained. Epidemiological data, comorbidities, sensitivity of the microorganisms to the antibiotic loaded in the cement spacer in the first revision surgery, time elapsed since an antibiotic was last administered until second revision procedure, functional shoulder status at last follow-up, and any complication were recorded.

**Results:**

Three out of the 22 cases (13.6%) presented positive cultures at the second-stage surgery. Periprosthetic tissue culturing detected the three positive culture cases in the second stage while the cement spacer sonication detected two and missed one. Considering periprosthetic tissue culturing as the standard procedure, the cement spacer sonication showed sensitivity at 66.6%. Recurrent infection over time was considered present in 3 patients; two of them had been previously diagnosed with a positive culture at the second stage (66.6%).

**Conclusions:**

A good number of patients (13.6%) present a positive culture at the second stage of the two-stage surgical procedure for infected shoulder arthroplasty, and those patients seem to be at high risk for recurrent infection. Periprosthetic tissue cultures have a higher sensitivity to detecting a positive culture at the second stage than cement spacer sonication.

## Background

Periprosthetic joint infection (PJI) still represents the most fearsome complication in shoulder arthroplasty. Despite reasonable good results having been reported with the one-stage treatment of deep infections in shoulder arthroplasty, the gold standard treatment for chronic PJI in shoulder remains the two-stage exchange using an antibiotic-loaded cement spacer between surgeries to maintain the tension of the soft tissue as well as to deliver the antibiotic [1–9].

Most of the knowledge of PJI management comes from hip and knee studies. Despite the intrinsic differences with the shoulder joint such as the shoulder being a limited weight-bearing joint, different organisms implicated in shoulder infections when compared to hip and knee, and the dependence of the shoulder to the rotator cuff muscles for function, the principles of treatment of shoulder PJI are adapted from those used in the hip and knee [7, 8, 10, 11]. Two-stage arthroplasty exchange still represents the standard way to manage PJI in hips and knees, but it has been reported that it may be successful in only 73% of the cases in knee arthroplasty and has a re-infection rate of 27% during follow-up [12]. It has also been documented, in hip and knee arthroplasty, that positive cultures may be still present in up to 20% of the cases undergoing two-stage procedure in the second stage. Furthermore, 63% of them may develop infection again over time. The rate of positive cultures was determined by culturing the sonication fluid of the cement spacer removed in the second surgery [13]. Two recent systematic reviews of two-stage revision for infected shoulder arthroplasty found a pooled success rate in 90.8 and 93.8% of the cases (ranging from 60 to 100%). However, little information is available relative to the rate of positive cultures in the second-stage surgery in shoulder arthroplasty and its meaning [14, 15].

In the same way, there are many studies supporting the use of the sonication fluid culturing to improve the diagnosis of infection in hip and knee joint replacement but there are few studies reporting the results of comparing the sensitivity of the culture of the explanted prostheses sonication fluid with the periprosthetic tissue culture in shoulder arthroplasty [13, 16–23].

The main objective of this study was to present the results of cement spacer sonication in the second stage of two-stage treatment of shoulder arthroplasty infection and to determine the rate of positive cultures in the second-stage surgery in shoulder arthroplasty and its meaning.

## Methods

It is a retrospective observational study that includes all deep infections of shoulder arthroplasties treated with a two-stage revision procedure from January 2006 to December 2014 in a tertiary hospital. Exclusion criteria included the cases where the cement spacer was not collected for sonication.

As defined by Zimmerli et al., infection was diagnosed if at least one of the following criteria was present: growth of the same microorganism in two or more cultures of synovial fluid or periprosthetic tissue, purulence of synovial fluid or at the implant site, acute inflammation on histopathological examination of periprosthetic tissue (periprosthetic joint infection is present if more than five neutrophils per high-power field in five high-power fields are observed from histological analysis of periprosthetic tissue at × 400 magnification), or presence of a sinus tract communicating with the prosthesis [24].

The first stage of treatment included the removal of the implants and all the cement, when present, the debridement of all affected soft tissue, and the implantation of a hand-made antibiotic-impregnated cement spacer. The second stage was done after antibiotic treatment was completed (minimum 6–8 weeks), the surgical wound was well healed, and the C-reactive protein value was normal after 2 weeks free of antibiotics. In the second stage, the cement spacer was removed and sent for sonication. At least four tissue cultures were obtained from the areas with a suspicion of poor soft tissue quality, and a new implant was put in. In all the cases, the new implant was cemented with antibiotic-loaded cement (tobramycin loaded). After intraoperative sampling, a broad-spectrum intravenous antibiotic regimen was started and continued until the result of the sonication and tissue cultures was known. Patients were classified as having a positive culture if the culture of the sonication fluid turned out to be positive and/or the culture of any periprosthetic tissue resulted positive.

The removed cement spacer was transported in a cylindrical polyethylene container. After covering about 90% of the spacer with a sterile saline solution (200–400 ml), the container was first vortexed for 30 s and then sonicated for 1 min at a frequency of 40 ± 5 kHz in a SM25E-MT Bransonic® ultrasound bath (Branson Ultrasonics Corporation, Geneva, Switzerland). After vortexing it again for 30 s, a 0.5-ml aliquot of the sonication fluid was used to inoculate both PolyViteX chocolate agar plates and Schaedler agar plates enriched with 5% sheep blood and onto thioglycolate broth. Between four and five intraoperative tissue specimens were collected in sterile vials and individually homogenized in 0.5 ml thioglycolate broth using a mortar and pestle. The tissue homogenate was inoculated onto the aforementioned media (0.1 ml per plate). The aerobic cultures were incubated at 37 °C in a 5% CO_2_-supplemmented atmosphere for 7 days and the anaerobic cultures at 37 °C for 14 days. The results were considered significant when the same microorganism was isolated in two or more cultures. One positive culture was also considered if it yielded the same microorganism as in the previous intraoperative cultures. The microbiological results were analyzed together with the presence of clinical signs and intraoperative findings of infection. The isolated microorganisms were identified by MALDI-TOF. Antimicrobial susceptibility testing was performed according to EUCAST criteria. Our standard operating procedure for samples collecting and transport assumes that once the tissue samples have been taken, they are immediately introduced into an individual sterile container for each sample, which is tightly closed and transported to the laboratory for analysis, with no possibility of air exposure during this process. The container is open in the laboratory and the samples are immediately plated.

The following data was recorded for all the patients included: age at the index surgery, diagnosis at the index surgery, type of shoulder prostheses at the index surgery, comorbidities, C-reactive protein (CRP) before the first revision surgery and before the second revision surgery, microorganisms isolated in the first revision surgery, the sensitivity of the microorganisms isolated in the first revision surgery to the antibiotic loaded in the cement spacer, the time elapsed since the termination of antibiotic treatment after the first stage until the second revision procedure, and the functional shoulder status before the first revision surgery and at last follow-up (minimum of 2 years) with the aid of the Constant Score and any complication during the follow-up period [25]. Values of C-reactive protein can be influenced by several different processes such as tissue damage, infection, inflammation, malignant neoplasia, and recent surgery. In elective shoulder arthroplasty, it has been demonstrated that CRP levels return to normal on the 14th day after surgery. In all the patients included, the elapsed time between surgeries was over 14 days [26].

All the patients included were followed for a minimum of 2 years (2–9 years).

Detailed data of all the patients are included in Table 1.

**Table 1:** Age, index diagnosis and procedure, infection signs, CRP before the first revision, and result of the cultures of the patients included

Patient	Age	Index diagnosis	Index procedure	Infection signs	CRP	Cultures 1st
1	79	CA	RSA	Pain + component loosening	.53	Negative
2	68	FS	RSA	Pain + component loosening	.33	Negative
3	79	AF	HA	Fistula	.67	*S. epidermidis*
4	69	AF	HA	Pus at surgery	.29	Negative
5	77	AF	HA	Fistula	NR	Negative
6	81	PA	HA	Fistula	21.4	*P. acnes*
7	47	PA	HA	Pus at surgery	1.7	Negative
8	65	CA	RSA	Fistula	1.9	*S. epidermidis*
9	76	AF	RSA	Fistula	1.04	*S. epidermidis + P. acnes*
10	73	AF	RSA	Pus at surgery	.16	Negative
11	86	AF	HA	Fistula	.6	Negative
12	62	AF	HA	Pus at surgery	1.27	*P. acnes*
13	55	PA	HA	Pain	.26	*P. acnes*
14	51	CA	RSA	Pus at surgery + dislocation	NR	*S. epidermidis*
15	45	AF	HA	Pain	.48	*P. acnes*
16	46	FS	RSA	Pus at surgery + dislocation	.38	Negative
17	70	AF	HA	Pain	NR	*S. epidermidis*
18	61	FS	RSA	Pus at surgery + dislocation	1.02	*P. acnes*
19	75	AF	RSA	Pus at surgery	.67	Negative
20	76	FS	RSA	Pain	NR	*E. faecalis + F. magna*
21	72	CA	RSA	Pus at surgery	11.6	*S. epidermidis + P. acnes*

*CA* cuff arthropathy, *FS* fracture sequelae, *AF* acute fracture, *PA* primary osteoarthritis, *RSA* reverse shoulder arthroplasty, *HA* hemiarthroplasty, *NR* not reported

### Statistical analysis

As the tables were built 2 by 2 (comorbidities for every one of covariates considered), the Fisher exact test and odds ratio (and its confidence interval) were calculated. Statistically significant results were taken at *p* values < 0.05. The differences in paired pre/post results were checked using the Wilcoxon signed-rank test. Power analysis was 0.5537577% due to the small number of infections. All analyses were performed with the R Statistical Package (R Foundation for Statistical Computing, Vienna, Austria; Version 3.0.2).

The study was approved by the Ethical Committee with number 2017/7413/I (CEIC-Parc de Salut Mar).

## Results

Twenty-one patients finally met inclusion criteria to be analyzed. There were 14 females and 7 males with a mean age of 67.5 (45–86). One patient underwent revision of the cement spacer, and a change was made for another cement spacer because of overt signs of infection, giving a total of 22 cases analyzed. Index surgery was performed because of trauma in 13 cases, rotator cuff arthropathy in 5 cases, and primary osteoarthritis in 3 cases. A reverse shoulder arthroplasty (RSA) was implanted in 12 cases and a hemiarthroplasty in 9 cases in the index surgery. According to Sperling et al., there were 2 acute, 4 subacute, and 16 late infections [8]. The infective organisms were *Propionibacterium acnes* in 8 cases, *Staphylococcus epidermidis* in 6, *Enterococcus faecalis* in 1, *Finegoldia Magna* in 1, and culture-negative in 8 cases. CRP was elevated in 7 out of the18 shoulders tested before the first revision surgery. The mean time elapsed from antibiotic cessation until the second revision surgery was 24.2 months (ranging from 2 to 210 months). In many instances, the patient postponed the second surgery for a long period of time due to the relative comfort the spacer provided.

Three out of the 22 cases (13.6%) presented positive cultures at the second-stage surgery. In the 2 of them, the same microorganism from the first stage was still present (one *S. epidermidis* and one *P. acnes*) while a microorganism not detected in the first stage was isolated (*Pseudomonas aeruginosa*) in the third case. Periprosthetic tissue culturing detected 3 positive culture cases in the second stage while cement spacer sonication detected 2 and missed 1. Considering periprosthetic tissue culturing as the standard procedure, the culturing of the sonication fluid showed sensitivity at 66.6%, a specificity of 100% (IC 97.3–100), a positive predictive value of 100% (IC 75–100), and a negative predictive value of 95% (IC 82.9–100).

Recurrent infection over time was considered present in 3 patients. Two of them had been previously diagnosed with a positive culture at the second stage (66.6%). Recurrent infection was significantly more common among patients having a positive culture during the second stage than among those patients having a negative culture (*p* = .038). The patients with a positive culture at the second stage underwent antibiotic treatment after the second-stage procedure but this did not prevent clinical failure. In patient number 3, antibiotic treatment was changed because the new strain of *S epidermidis* isolated was resistant to rifampin, then rifampin was substituted with cotrimoxazole. In patient number 11, with negative cultures at the first step exchange, we empirically used ciprofloxacin plus rifampin and only ciprofloxacin at the second step when a *Pseudomonas* was isolated in both tissue and sonication cultures. In patient number 21, the treatment with amoxicillin was not changed. In this patient, the first spacer was changed for a new one while waiting for the second step exchange because of new clinical and analytical signs of infection. Only one tissue sample was positive to *P. acnes*, because the patient was still under targeted antibiotic treatment. Despite this, we cannot discard bacterial contamination. Two of the clinical failures underwent further surgery (a two-stage component exchange), and the third one underwent suppressive antibiotic treatment due to comorbidities and advanced age.

Considering recurrent infection as the end point, the periprosthetic tissue cultures could detect 2 and missed 1 and the cement spacer sonication could detect 1 and missed 2.

All the cement spacers were hand-made with tobramycin. In 6 cases, the tobramycin was ineffective against the microorganisms isolated in the index revision but no significant differences could be found between spacers with effective antibiotic versus those with ineffective antibiotic (*p* = 0.155).

The index diagnosis of a trauma case significantly increased the risk of a positive culture at the second stage (*p* = 0.01) with an odds ratio of 1167.

The comorbidities analyzed (hypertension in 15 patients, diabetes in 5 patients, and hypothyroidism in 3 patients), the time elapsed between the end of antibiotic treatment, and the second-stage surgery did not have an influence on the presentation of a positive culture at the second stage or on clinical failure.

The Constant Score significantly improved from 16.3 to 34.1 (*p* = .026) after the two-stage procedure, mainly because of pain improvement. Forward elevation and external and internal rotation as well as strength did not significantly improve after treatment as assessed with the Constant Score. Range of motion in forward elevation increased from 46.1° (SD 31.2°) to 78.2° (SD 47.5°), but it did not reach significance (*p* = .053).

Nine patients out of the 21 included (42.8%) developed some complication during follow-up that included persistent pain, dislocation of RSA, humeral component loosening, a periprosthetic fracture, axillar nerve injury during revision surgery, and infection recurrence. Detailed data of all the patients included is in Table 2.

**Table 2:** Number of tissue cultures obtained, germen from tissue cultures and from cultures of sonication fluid, recurrence of infection, and complications at follow-up

Patient	Number of tissue cultures	Microorganism tissue cultures	Microorganism sonication	Persistent infection	Complications
1	5	Negative	Negative	No	Persistent pain
2	4	Negative	Negative	No	None
3	4	*S. epidermidis*	*S. epidermidis*	No	Dislocation
4	5	Negative	Negative	No	None
5	5	Negative	Negative	No	None
6	5	Negative	Negative	No	None
7	5	Negative	Negative	No	None
8	4	Negative	Negative	No	None
9	5	Negative	Negative	No	None
10	5	Negative	Negative	No	Nerve injury
11	5	*P. aeruginosa*	*P. aeruginosa*	Yes	Fistula
12	6	Negative	Negative	No	None
13	5	Negative	Negative	No	None
14	5	Negative	Negative	No	Humeral loosening
15	5	Negative	Negative	No	None
16	5	Negative	Negative	Yes	Fistula
17	6	Negative	Negative	No	None
18	5	Negative	Negative	No	Dislocation
19	5	Negative	Negative	No	None
20	5	Negative	Negative	No	Periprosthetic fracture
21	10	*P. acnes*	Negative	Yes	Fistula

## Discussion

The two-stage treatment of infected shoulder arthroplasty may be successful in > 90% of the cases; however, a number of patients present recurrent infection over time. The results of the present study provide evidence that a positive culture may be present in the second stage of two-stage treatment of an infection. Sonication of the cement spacer removed at the second surgery has less sensitivity than periprosthetic tissue cultures in detecting a positive culture at the second stage.

Two-stage treatment of an infected arthroplasty remains the standard treatment for patients with a chronic infection. Despite that, the success of two-stage treatment can be as low as in 73% of the patients in knee replacement [12]. Sorli et al. determined that, among the 55 patients, consisting of 37 knees, 17 hips, and 1 shoulder treated with two-stage treatment of an infected arthroplasty, 20% of them presented a subclinical infection at the time of the second surgery and 63% of that 20% developed a recurrence of the infection over time. Of those without a subclinical infection in the second stage of a two-stage surgery, only 25% went on to develop an infection over time [13]. In the infected shoulder arthroplasty, the recurrence of infection has been reported to be from 19 to 37% of the patients treated with two-stage treatment [2, 3, 5–9]. In the present study, 3 out of 21 patients (14.3%) presented recurrent infection. At the time of the second surgery, 3 patients presented a positive culture with positive periprosthetic tissue cultures and/or positive cultures of the sonication of the cement spacer. Two of the 3 patients with a positive culture at the second stage developed a recurrent infection over time (66.6%) compared to one of the 18 patients without a positive culture (5.5%). The 3 patients with a positive culture at the second stage received specific antibiotic treatment for 12 weeks, but it did not prevent recurrence of the infection. Zhang et al. found persistent infection in 22% of the patients treated with a two-stage treatment by doing an open biopsy during staged treatment. This percentage was even higher (38%) in patients with a *P. acnes* infection [27]. It seems that a number of patients present a positive culture at the second stage of the two-stage procedure and, based on the results of the present study, these patients seem to be at high risk of recurrent infection, but studies with a larger number of patients included are needed before establishing strong conclusions on that.

Trampuz et al. published that the sensitivity of the sonicate fluid cultures (78.5%) was significantly higher than the sensitivity of the periprosthetic tissue cultures (60.8%) in infected hip and knee prostheses [22]. After that, many studies support the use of the sonicate fluid cultures to detect infection with a higher sensitivity than periprosthetic tissue cultures, especially if the patients are undergoing antibiotic treatment [13, 16–18, 20, 21]. Pipper et al. also demonstrated that the sonicate fluid culture was more sensitive than the periprosthetic tissue cultures in prosthetic shoulder infection (66.7 vs 59.5%) [19]. However, the differences in sensitivity between periprosthetic cultures and the culturing of the sonication fluid seem to depend on the number of periprosthetic tissue cultures obtained. In the same study, Trampuz et al. determined that the sensitivity of tissue cultures increased from 50.0 to 54.1% to 66.7 to 72.7% if the number of cultures obtained increased from two or three to four or five or more [22]. Many of the studies reporting the greater sensitivity of the sonication fluid culture included patients with less than 3 periprosthetic tissue cultures. In the study by Pipper et al. in shoulder prostheses, the exclusion criterion was patients with less than two tissue cultures [19]. Recently, Van Diek et al. reported the lower sensitivity of the sonication fluid analysis (0.47) when compared to the sensitivity of the tissue culture (0.68) among 252 consecutive revision arthroplasty cases. In this study, the exclusion criterion was less than six tissue cultures [28]. Grosso et al. also reported no significant benefits of implant sonication compared to intraoperative cultures in diagnosis of PJI in 53 revision shoulder arthroplasties with a mean of 4.5 specimens for culture [23]. In the present study, all the cases included had at least four tissue periprosthetic cultures. Considering periprosthetic tissue culturing as the gold standard, the culturing of sonication fluid has resulted in a sensitivity of only 66.6%. Periprosthetic tissue culturing detected the three positive cultures at the second stage, and the culturing of the cement spacer sonication missed one (the *P. acnes*). Therefore, cement spacer sonication is unnecessary if at least four periprosthetic tissue cultures are obtained for the diagnosis of a positive culture in the second stage of a two-stage treatment of an infected shoulder arthroplasty. In the present study, no patient was undergoing antibiotic treatment. Thus, further studies are needed to determine whether the use of the cement spacer sonication is of any help under this circumstance in shoulder arthroplasty infection.

It is also remarkable that the number of culture-negative PJI in the shoulder is higher than that reported for hip and knee surgery. Berbari et al. determined that there was a 7% of culture-negative PJI in hip and knee PJI among 897 episodes of PJI. Prior antibiotic therapy and biofilm-producing microorganisms were among the reasons for culture-negative PJI [29]. In the present study, 36% of the patients presented a culture-negative PJI. Recently, Buchalter et al., in a series of 19 patients undergoing two-stage revision for an infected shoulder arthroplasty, also reported a high number of culture-negative PJI (31.5%) [2]. In all likelihood, the fact that *P. acnes* is involved in a higher proportion in shoulder PJI rather than in hip and knee PJI can make this culture-negative difference greater, but further studies are needed before asserting that.

The clinical outcome of two-stage surgery for infected shoulder arthroplasty provides limited results and a high number of complications. In the present study, the final mean absolute Constant Score was of only 34.1 even though a significant improvement in the Constant Score was noted. The mean forward elevation did not significantly improve as measured with the Constant Score, and there was a limited non-significant improvement from 52° to 78.2° when measured with degrees. George et al. and Nelson et al. failed to find significant differences between one-stage and two-stage surgery for PJI in shoulder arthroplasty in systematic reviews. However, the one-stage seems to bring about better outcomes [14, 15]. Complications after two-stage treatment have been reported to be high, 36% reported by Jacquot et al., in a French multicenter study [5], and 42% reported by Buchalter et al., in a series of 19 patients [2]. Nine of the 21 patients included in the present study presented some complication (42,8%) including a dislocation of RSA, a periprosthetic fracture, axillar nerve injury during revision surgery, and recurrent infection.

Among the limitations of the study are the inherent limitation of its retrospective nature and the small sample size. Among the strengths, it represents a study with the largest series of cement spacer sonication in shoulder surgery.

## Conclusions

A good number of patients (13.6%) present a positive culture at the second stage of the two-stage surgical procedure for infected shoulder arthroplasty, and those patients seem to be at high risk for recurrent infection.

Whenever four or more periprosthetic tissue cultures are obtained, the sonication of the cement spacer fluid seems to be of no benefit to detect a positive culture at the second stage of two-stage surgery for PJI in shoulder arthroplasty.

## Abbreviations

CRP: C-reactive protein
PJI: Periprosthetic joint infection
RSA: Reverse shoulder arthroplasty
